# Supplementary material for: Properties of repression condensates in living Ciona embryos
Source: Nat Commun. 2021 Mar 10;12:1561. doi: 10.1038/s41467-021-21606-5 (PMC7946874; doi:10.1038/s41467-021-21606-5)
Supplement: Supplementary file 1 — Supplementary Information [file 41467_2021_21606_MOESM1_ESM.pdf]

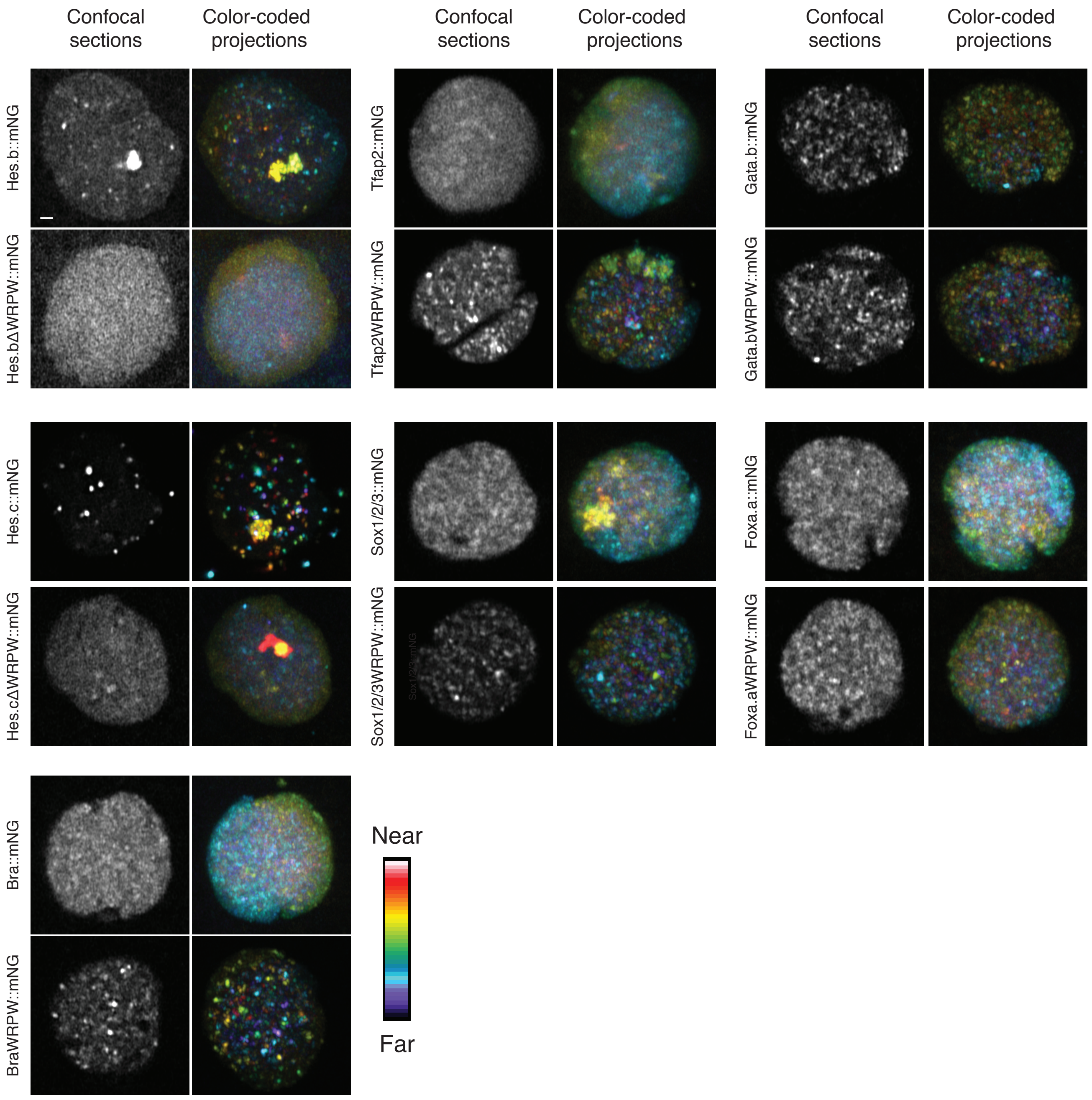

Supplementary Figure 1: The WRPW motif is required for and can induce transcription factors to from puncta.

Single confocal sections and color-coded projections of single nuclei expressing transcription factors fused to mNg with or without a C-terminal WRPW domain. Scale bar = 1 µm. All transgenes were expressed using pSP *Sox1/2/3*> plasmids. All images are representative of >3 biological replicates.

a

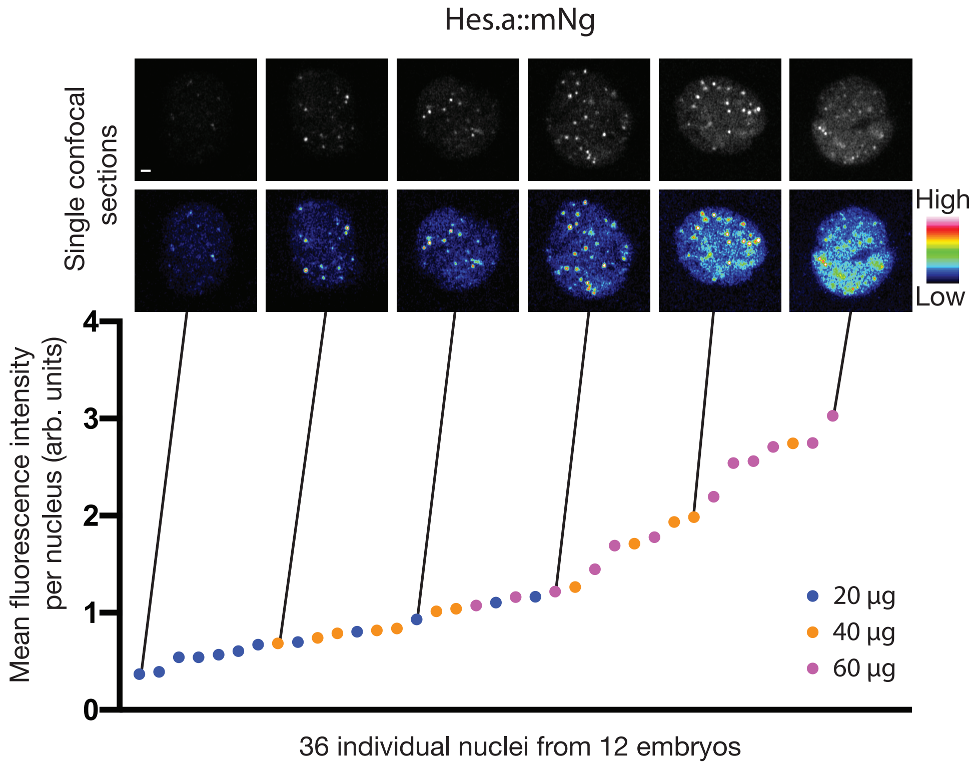
Hes.a::mNg
Single confocal sections
High
Low
Mean fluorescence intensity per nucleus (arb. units)
4
3
2
1
0
20 µg
40 µg
60 µg
36 individual nuclei from 12 embryos

b

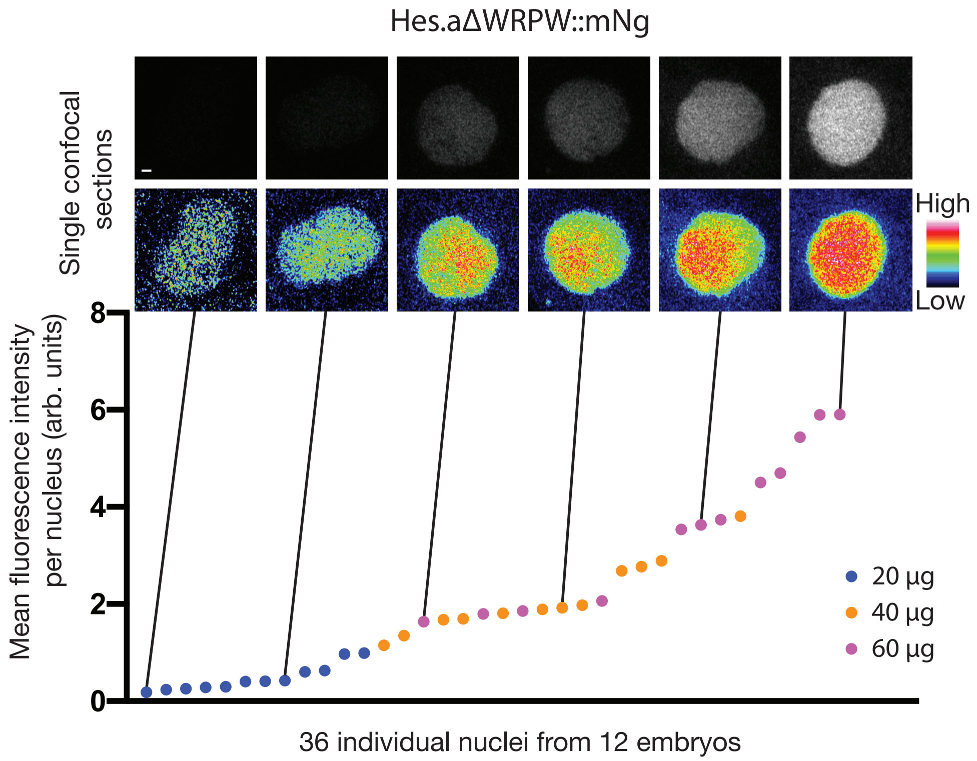
Hes.aΔWRPW::mNg
Single confocal sections
High
Low
Mean fluorescence intensity per nucleus (arb. units)
8
6
4
2
0
20 µg
40 µg
60 µg
36 individual nuclei from 12 embryos

Supplementary Figure 2: Nuclear distributions of Hes.a at different concentrations.

a, Mean fluorescent intensity was calculated from central confocal sections of *Ciona* nuclei expressing Hes.a ::mNG. Confocal images are shown in grayscale and with the indicated look up table. Fluorescent intensity from all grayscale images are directly comparable. Fluorescence measurements are ranked from lowest to highest and the electroporation condition performed is indicated by the color of the circle. **b,** Same as **a** but for Hes.a aΔWRPW::mNG. Scale bar = 1 µm. All transgenes were expressed using pSP *Sox1/2/3*> plasmids. Source data are provided as a Source Data file.

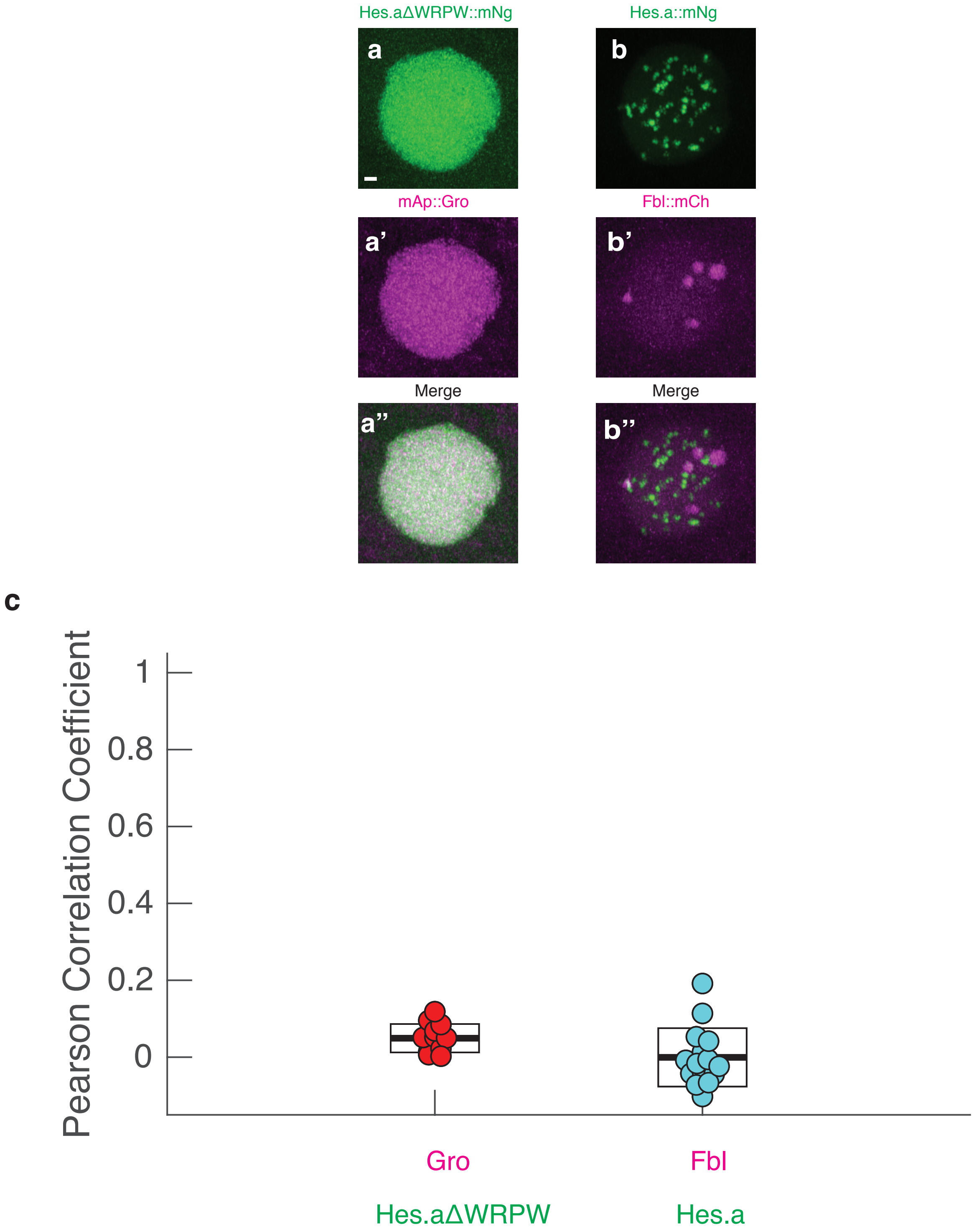

Hes.aΔWRPW::mNg
Hes.a::mNg
a
b
mAp::Gro
Fbl::mCh
a'
b'
Merge
Merge
a''
b''
c
Pearson Correlation Coefficient
1
0.8
0.6
0.4
0.2
0
Gro
Fbl
Hes.aΔWRPW
Hes.a

Supplementary Figure 3: Hes.a colocalization analysis.

a, Maximum intensity confocal projections of *Ciona* nuclei expressing Hes.aΔWRPW::mNG. Scale bar = 1 µm. **a**’ As **a** but for mAp::Gro. **a**’’, Merged green and red channels for **a** and **a**’. **b**-**b**’’, Same as **a**-**a**’’ but for Hes.a::mNg and Fbl::mCh. **C**, Person correlation coefficients of the experiments shown in **a**-**b**’’. Boxes display the mean as the central line and the limits of the box denote standard deviations. All transgenes were expressed using pSP *Sox1/2/3*> plasmids. All images are representative of >3 biological replicates. Source data are provided as a Source Data file.

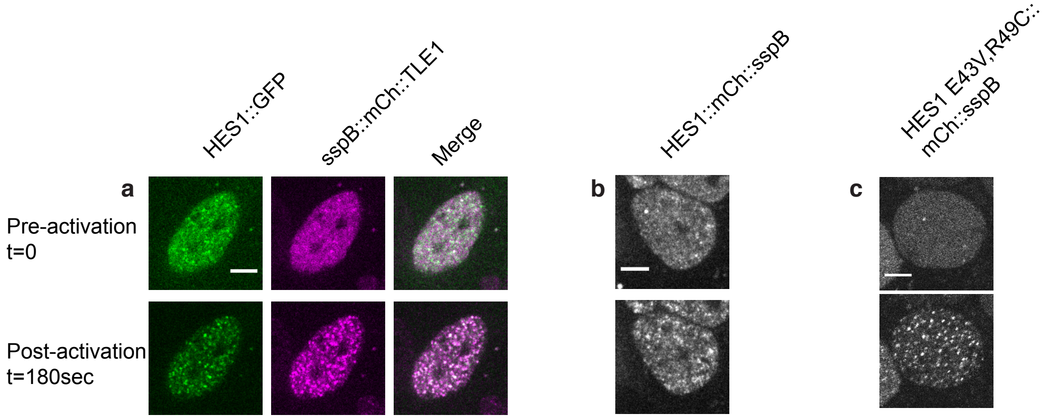

Supplementary Figure 4: Human HES1 and TLE corelets show light inducible aggregation and phase separation.

a, A single human cell nucleus expressing HES1::GFP and sspB::mCh-TLE1 before and after light activation. **b**, Wildtype HES1-mCh-sspB behavior before and after light induced binding of corelets. **c**, Same as **b,** but for the DNA binding domain mutant of HES1. Scale bar = 5 µm. All images are representative of >3 biological replicates.

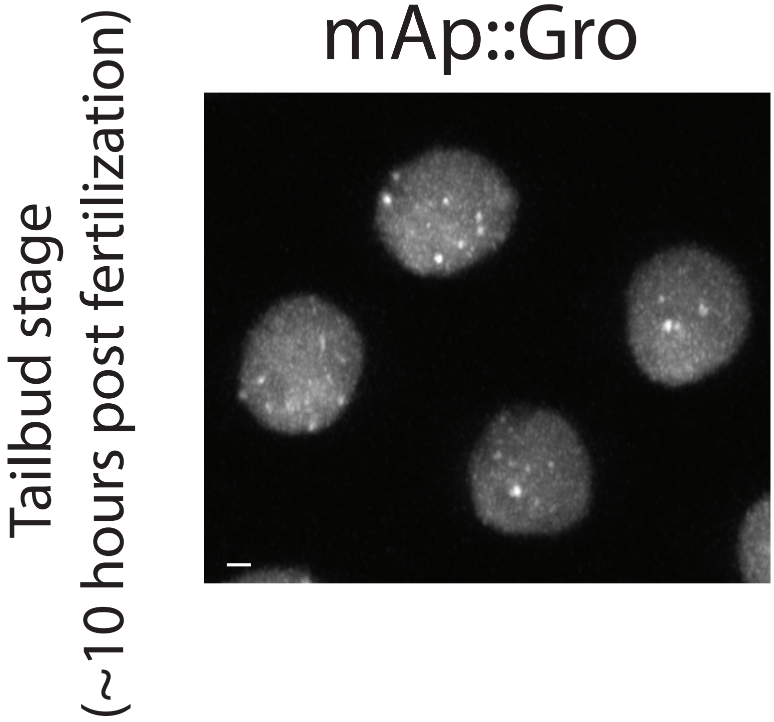

Supplementary Figure 5. Gro forms nuclear puncta in tailbud stage *Ciona* embryos A maximum intensity confocal projection of a region of tail epidermis in tailbud stage *Ciona* embryos. Scale bar = 1 µm. mAp::Gro was expressed using pSP *Sox1/2/3*> plasmids. All images are representative of >3 biological replicates.

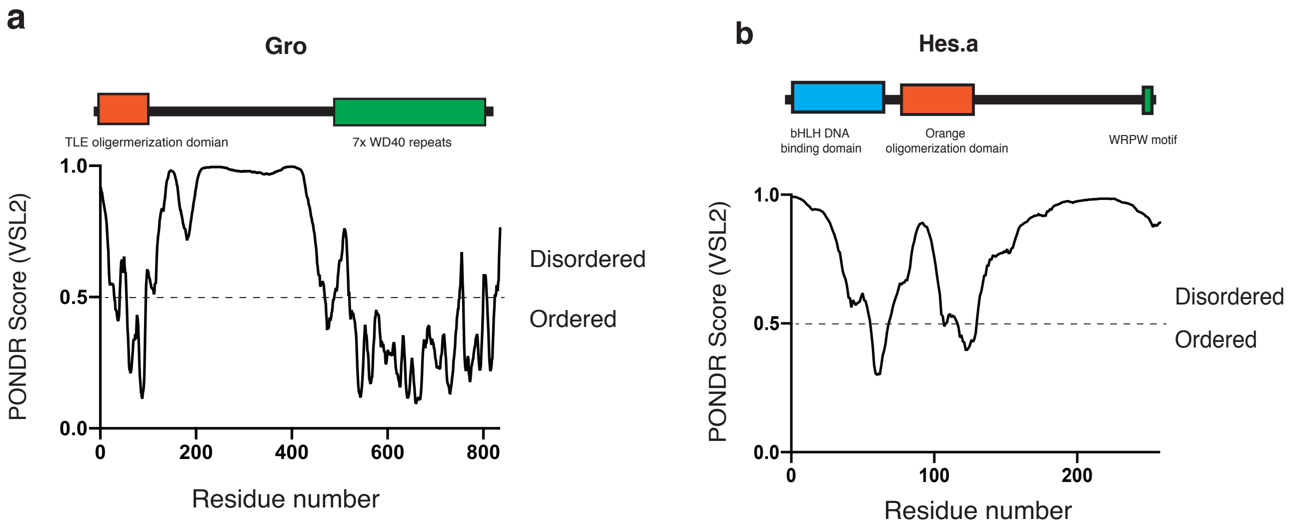

Supplementary Figure 6: Primary structure of Groucho and Hes.a proteins.

a, For Gro, annotated domains are indicated as well as a PONDR score to show predictions of ordered or disordered structure throughout the protein. **b,** Same analysis as **a** but for Hes.a. Source data are provided as a Source Data file.

Supplementary Table 1:

Name in text	Synonyms	KH (2012) identity	ORF cDNA primers (not including noncomplimentary sequences or stop codons)
Sox1/2/3	SoxB1	KH.C1.99	ATGTTAACCGTTGACCACAAC, CATATGCGCAGGCATGTGCA
Hes.a	E(spl)/hairy-a	KH.C1.159	ATGCCTGTTGAACGAAGAAT, TAATCCCCAAGGGCGCCACA
Hes.b	E(spl)/hairy-b	KH.C3.312	ATGAAGACGGTAATGACTCC, CCATGGTCTCCATACTGGAT
Hes.c	E(spl)/hairy-c	KH.L34.9	ATGTGTGACGTCATGCAAC, ATCGCCCACCAAATCTGTTTC
Snai	Snail	KH.C3.751	ATGACCTCCGTCGAGCCCAT, GGATGCTGTCTTGCGTTGTG
Gro	Groucho2, TLE	KH.L96.11	ATGTTCCCAAACAGACCCCA, TGCCATAACTTCGTAAA
Cdyl		KH.L126.11	ATGACAAGAAGTAAAAAACAAG, AGAAATAAAATCATGAG
Fbl	Fibrillarin	KH.C4.401	ATGGGACGTCCAGGTTTTTCTCC, TTTCTTGTTCTTTGGTG
Hp1a	Cbx1	KH.C1.912	ATGGGAAAAAAGAAAATGGA, GCTCTGGTCGTCTGGGGAAG
Bra	Brachyury, T	KH.S1404.1	ATGGCGCTAATAGAGCATGG, CAAAGAAGGTGGCGTAAGCG
Tfap2-r.b	AP-2-like2	KH.C7.43	ATGAGTGATATTCGAATTCTG, TTTGTCGTTTTTGTCGGAAA
Gata.b	Gata-B	KH.S696.1	ATGATGCCAACAAGTAGCG, GCCCATTGCGTGTACCATAC
Foxa.a	FoxA-a	KH.C11.313	ATGATGTTGTCGTCTCCACC, GCTTGCTGGTACGCACCCTG
HES1 (human)		N/A	ATGCCAGCTGATATAATGGAG, GTTCCGCCACGGCCTCCA
TLE1 (human)		N/A	TTCCCGCAGAGCCGGCAC, GTAGATGACTTCATAGAC
