# Supplementary material for: Properties of repression condensates in living Ciona embryos
Source: Nat Commun. 2021 Mar 10;12:1561. doi: 10.1038/s41467-021-21606-5 (PMC7946874; doi:10.1038/s41467-021-21606-5)
Supplement: Supplementary file 3 — Description of Additional Supplementary Files [file 41467_2021_21606_MOESM3_ESM.pdf]

Description of Additional Supplementary Files

Supplementary Movie 1: Fibrillarin dynamics during development.

Maximum intensity confocal projection of Fbl::mNg (in green) and H2b::mCh (in magenta) throughout a full mitosis. Time is in minutes relative to metaphase. The video is representative of >3 biological replicates.

Supplementary Movie 2: The fusion of 2 fibrillarin droplets.

Maximum intensity confocal projection of the fusion of 2 Fbl::mNg droplets. The video is representative of >3 biological replicates.

Supplementary Movie 3: Hes.a dynamics during development.

Maximum intensity confocal projection of Hes.a::mNg (in green) and H2b::mCh (in magenta) throughout a full mitosis. Time is in minutes relative to metaphase. The video is representative of >3 biological replicates.

Supplementary Movie 4: Hes.a DNA binding mutant dynamics during development

Maximum intensity confocal projection of Hes.a E22v,R28C::mNg (in green) and H2b::mCh (in magenta) throughout a full mitosis. Time is in minutes relative to metaphase. The video is representative of >3 biological replicates.

Supplementary Movie 5: FRAP of Hes.a droplets

Maximum intensity confocal projection of *Ciona* nucleus electroporated with Hes.a::mNg. The region indicated by the white rectangle was photobleached. The video is representative of >3 biological replicates.

Supplementary Movie 6: FRAP of Hes.a DNA binding mutant droplets

Maximum intensity confocal projection of *Ciona* nucleus electroporated with Hes.a E22v,R28C::mNg. The region indicated by the white rectangle was photobleached. The video is representative of >3 biological replicates.

Supplementary Movie 7: The inability of Hes.a droplets to fuse.

Maximum intensity confocal projection of the fusion of 2 Hes.a::mNg droplets. The video is representative of >3 biological replicates.

Supplementary Movie 8: The fusion of 2 Hes.a droplets after treatment with latrunculin a.

Maximum intensity confocal projection of the fusion of 2 Hes.a::mNg droplets in an embryo treated with latrunculin a. The video is representative of >3 biological replicates.

Supplementary Movie 9: The fusion of 2 Hes.a E22V,R28C droplets.

Maximum intensity confocal projection of the fusion of 2 Hes.a E22v,R28C::mNg droplets. The video is representative of >3 biological replicates.

Supplementary Movie 10: HES1 TLE corelet colocalization.

A single human cell nucleus expressing HES1::GFP (in green) and sspB::mCh-TLE (in magenta) Time is in seconds after activation. Scale bar = 5 µm. The video is representative of >3 biological replicates.

Supplementary Movie 11: HES1 corelet aggregation.

A single human cell nucleus expressing wildtype HES1-mCh-sspB Time is in seconds after activation. Scale bar = 5 µm. The video is representative of >3 biological replicates.

Supplementary Movie 12: HES1 E43V,R49C mutant corelet aggregation.

A single human cell nucleus expressing HES1E43V,R49C-mCh-sspB Time is in seconds after activation. Scale bar = 5 µm. The video is representative of >3 biological replicates.
