# Supplementary material for: Properties of repression condensates in living Ciona embryos
Source: Nat Commun. 2021 Mar 10;12:1561. doi: 10.1038/s41467-021-21606-5 (PMC7946874; doi:10.1038/s41467-021-21606-5)
Supplement: Supplementary file 16 — Reporting Summary [file 41467_2021_21606_MOESM16_ESM.pdf]

nature research

Corresponding author(s): Michael Levine

Last updated by author(s): Dec 21, 2020

Reporting Summary

Nature Research wishes to improve the reproducibility of the work that we publish. This form provides structure for consistency and transparency in reporting. For further information on Nature Research policies, see Authors & Referees and the Editorial Policy Checklist .

Statistics

For all statistical analyses, confirm that the following items are present in the figure legend, table legend, main text, or Methods section.

n/a	Confirmed	
☐	☒	The exact sample size (*n*) for each experimental group/condition, given as a discrete number and unit of measurement
☐	☒	A statement on whether measurements were taken from distinct samples or whether the same sample was measured repeatedly
☒	☐	The statistical test(s) used AND whether they are one- or two-sided<br>*Only common tests should be described solely by name; describe more complex techniques in the Methods section.*
☐	☒	A description of all covariates tested
☐	☒	A description of any assumptions or corrections, such as tests of normality and adjustment for multiple comparisons
☐	☒	A full description of the statistical parameters including central tendency (e.g. means) or other basic estimates (e.g. regression coefficient) AND variation (e.g. standard deviation) or associated estimates of uncertainty (e.g. confidence intervals)
☒	☐	For null hypothesis testing, the test statistic (e.g. *F*, *t*, *r*) with confidence intervals, effect sizes, degrees of freedom and *P* value noted<br>*Give P values as exact values whenever suitable.*
☒	☐	For Bayesian analysis, information on the choice of priors and Markov chain Monte Carlo settings
☒	☐	For hierarchical and complex designs, identification of the appropriate level for tests and full reporting of outcomes
☐	☒	Estimates of effect sizes (e.g. Cohen's *d*, Pearson's *r*), indicating how they were calculated

Our web collection on statistics for biologists contains articles on many of the points above.

Software and code

Policy information about availability of computer code

Data collection	Zeiss microscopy software, ZEN Version 2.3 and 2.6
Data analysis	Published data analysis code described in the methods was used in this study

For manuscripts utilizing custom algorithms or software that are central to the research but not yet described in published literature, software must be made available to editors/reviewers. We strongly encourage code deposition in a community repository (e.g. GitHub). See the Nature Research guidelines for submitting code & software for further information.

Data

Policy information about availability of data

All manuscripts must include a data availability statement. This statement should provide the following information, where applicable:

- Accession codes, unique identifiers, or web links for publicly available datasets
- A list of figures that have associated raw data
- A description of any restrictions on data availability

Unprocessed images and other materials can be provided upon request.

Field-specific reporting

Please select the one below that is the best fit for your research. If you are not sure, read the appropriate sections before making your selection.

☒ Life sciences ☐ Behavioural & social sciences ☐ Ecological, evolutionary & environmental sciences

For a reference copy of the document with all sections, see nature.com/documents/nr-reporting-summary-flat.pdf

Life sciences study design

All studies must disclose on these points even when the disclosure is negative.

Sample size	For quantifications reported 3 biological replicates were performed. No sample-size calculations were performed. For all experiments, similar results were observed from >3 biological replicates.
Data exclusions	For the FRAP analysis some data points where the droplet temporarily moved out of focus were excluded.
Replication	All experiments were repeated with at least 3 biological replicates and could be observed in hundreds of embryos For the key experiments such as the puncta formation of Hes.a, the experiments have been repeated over 20 times. All replication attempts were successful.
Randomization	Randomization of samples was not feasible or necessary for this study as they involved live imaging of fast developing embryos and were performed by a single investigator.
Blinding	Blinding of samples was not feasible or necessary for this study as they involved live imaging of fast developing embryos and were performed by a single investigator.

Reporting for specific materials, systems and methods

We require information from authors about some types of materials, experimental systems and methods used in many studies. Here, indicate whether each material, system or method listed is relevant to your study. If you are not sure if a list item applies to your research, read the appropriate section before selecting a response.

Materials & experimental systems

n/a	Involved in the study	
☒	☐	Antibodies
☐	☒	Eukaryotic cell lines
☒	☐	Palaeontology
☐	☒	Animals and other organisms
☒	☐	Human research participants
☒	☐	Clinical data

Methods

n/a	Involved in the study	
☒	☐	ChIP-seq
☒	☐	Flow cytometry
☒	☐	MRI-based neuroimaging

Eukaryotic cell lines

Policy information about cell lines

Cell line source(s)	HEK293 (Provided from Marc Diamond UTSW)
Authentication	Cell lines were not authenticated.
Mycoplasma contamination	Cell lines were not directly screened for mycoplasme
Commonly misidentified lines (See ICLAC register)	None

Animals and other organisms

Policy information about studies involving animals; ARRIVE guidelines recommended for reporting animal research

Laboratory animals	None
Wild animals	Wild Ciona intestinalis obtained from commercial suppliers were used for this study. Ciona were shipped from San Diego to Princeton, held in an aquarium until required, gametes were removed by surgical extraction and Ciona were killed by freezing.
Field-collected samples	None
Ethics oversight	These animals are invertebrates and do not require ethics oversight.

Note that full information on the approval of the study protocol must also be provided in the manuscript.
